# Unraveling clinical outcomes of long-term cART treatment in HIV-1 patients with or without the Brazilian GWGR motif in the V3 loop

**DOI:** 10.1590/S1678-9946202466038

**Published:** 2024-07-08

**Authors:** Victor Ângelo Folgosi, Shirley Vasconcelos Komninakis, Luciano Lopes, Mariana Amélia Monteiro, Tatiane Assone, Luiz Augusto Marcondes Fonseca, Wilson Domingues, Pedro Domingos Leite, Jefferson Russo Victor, Jorge Casseb

**Affiliations:** 1 Universidade de São Paulo, Faculdade de Medicina, Instituto de Medicina Tropical de São Paulo, Laboratório de Investigação Médica (LIM-56), São Paulo, São Paulo, Brazil; 2 Universidade Federal de São Paulo, Laboratório de Retrovirologia, São Paulo, São Paulo, Brazil; 3 Universidade Federal de São Paulo, Departamento de Informática em Saúde, Divisão de Bioinformática e Ciência de Dados em Biologia, São Paulo, São Paulo, Brazil; 4 Universidade Santo Amaro, Programa de Pós-Graduação em Ciências da Saúde, São Paulo, São Paulo, Brazil

**Keywords:** HIV-1, ART, Subtype B, GWGR signature, Brazil

## Abstract

The presence of genetic mutations in HIV poses a significant challenge, potentially leading to antiretroviral resistance and hampering therapeutic development. The Brazilian population has presented variations in the HIV envelope V3 loop gene, especially the GWGR motif. This motif has been linked to reduced transmission potential and slower CD4+ T cell decline. This study aimed to assess clinical outcomes in patients with HIV-1 infected with strains containing the GWGR motif compared with those without it during long-term cART. A cohort of 295 patients with HIV was examined for the GWGR motif presence in the V3 loop. A total of 58 samples showed the GWGR signature, while 237 had other signatures. Multifactorial analyses showed no significant differences in demographic characteristics, CD4+ cell count, AIDS progression, or mortality between GWGR carriers and others. However, the mean interval between the first positive HIV test and the initial AIDS-defining event was more than two times longer for women carrying the GWGR signature (p = 0.0231). We emphasize the positive impact of cART on HIV/AIDS treatment, including viral suppression, CD4+ cell preservation, and immune function maintenance. Although no significant differences were found during cART, residual outcomes reflecting adherence challenges were observed between diagnosis and the first AIDS-defining event. The previously described outcomes, highlighting statistically significant differences between individuals carrying the GPGR motif compared with those with the Brazilian GWGR motif, may be directly linked to the natural progression of infection before advancements in cART. Presently, these physicochemical aspects may no longer hold the same relevance.

## INTRODUCTION

Since 2004, a broad range of comprehensive programs for preventing, caring for, and treating HIV has been implemented in over 30 low- and middle-income countries worldwide. Starting in 2016, these initiatives facilitated access to antiretroviral treatment (ART) for approximately 19.5 million individuals living with HIV (PLHIV), regardless of immune and virological status, to combat HIV-1^1^. Brazil’s political commitment in 1996 ensured the universal distribution of combined therapy, effectively fighting prejudice and dispelling the association of HIV with death. The rights of PLHIV support access to free treatment was achieved via the efforts of social movements combined with scientific evidence^2^. This achievement markedly improved PLHIV’s quality of life and substantially mitigated virus transmission on a global scale^3,4^.

Despite the substantial advancements in the development of therapies and prevention strategies for HIV, the virus continues to pose significant challenges to global public health^5,6^. Research efforts related to HIV not only focus on optimizing existing antiretroviral therapies but also on identifying new targets and approaches to prevent and treat the infection^7^. Furthermore, numerous researchers have dedicated their efforts to investigate the temporal evolution of the virus, considering its genetic diversity and adaptive capabilities in different environments and treatments^8-11^.

The HIV-1, known for its remarkable genetic variability, exhibits cellular tropism via complex interactions involving the major glycoproteins encoded by the env gene, namely gp41 and gp120, and cellular receptors^3,12^. The envelope glycoprotein (gp120) is highlighted in the virus-host interaction, especially in its affinity with the CD4 molecule expressed on the surface of human T lymphocytes^13,14^. After the initial binding of gp120 to the CD4 molecule, conformational changes occur in the envelope, followed by the involvement of chemokine receptors, primarily CCR5 (C-C chemokine receptor type 5) or CXCR4 (C-X-C chemokine receptor type 4), essential for the efficient entry of the virus into the host cell^3,12-14^.

The hypervariable central region plays a crucial role in the cellular fusion process, with emphasis on the V3 loop present in gp120. This loop plays an essential role in binding to the host CCR5 coreceptor, facilitated by a tetrapeptide motif that exhibits relative conservation (GPGR)^15,16^. This motif spans amino acids 312-315 of gp120 and is located in the crown of the V3 loop^17^. Despite the relative degree of conservation of GPGR, this motif presents substantial variability due to the need to maintain the functional role of the V3 loop. Besides GPGR, found in the HIV-1 reference strain, the GPGQ and GPGK motifs are massively found in HIV-1 strains^18^.

In the Brazilian context, four distinct subtypes (B, C, D, and F) have been delineated^19^, along with recombinant forms B/F and B/C, all falling within group M^20-22^. Furthermore, molecular analyses have revealed the coexistence of two distinct strains of subtype B HIV-1 in Brazil, which are genetically and antigenically different. One strain shows a GPGR motif in the crown of the V3 loop, resembling HIV-1 isolates originating from the USA and Europe^22-24^. Meanwhile, the other is recognized as a Brazilian variant, distinguished by a unique signature in the crown of the V3 loop, denoted as GWGR. This variant is characterized by substituting proline with tryptophan at position 313 (P313W) of the HIV-1 envelope^8-10,25^. Note that the GWGR variant accounts for 17 to 50% of subtype B HIV-1 infections in Brazil^8-10,26^. This distinctive signature has also been sporadically identified in approximately 23 countries^9,11^.

Some clinical research suggested that the HIV-1 strains GWGR motif may confer lower pathogenicity compared with other more prevalent signatures in the USA and Europe, implying a reduction in AIDS-defining events^8,27^. The GWGR motif uniquely relies on the CCR5 co-receptor^10^, and R5 variants that employ CCR5 are generally associated with a slower progression to AIDS^28,29^. Additionally, the GWGR motif promotes or is correlated with a higher affinity for neutralizing antibodies produced against the V3 region, contributing to a slower disease progression^30,31^. On the other hand, HIV-1 strains with GPGR, GPGQ, and GPGK motifs in their V3 crown can escape from neutralizing antibodies^32^ and resist fusion inhibitor drugs, conferring the most pathogenic clinical course^18^.

Patients infected with HIV-1 strains containing the GWGR motif exhibit a longer interval between the first positive test for HIV-1 and the first defining AIDS event, with this interval potentially being up to three times longer than the interval observed in patients infected with HIV-1 strains containing the GPGR motif^8,27,31^. Additionally, a higher peripheral CD4+ T cell count was observed. These individuals exhibited a lower viral load, higher levels and avidity of anti-V3 antibodies, and a greater propensity for more extended asymptomatic periods after infection^8,31^. Furthermore, women infected with HIV-1 Brazilian strain showed a significantly lower risk of hospitalization compared with those infected with the GPGR signature^8,27^.

However, despite numerous studies investigating the potential implications of HIV-1 strains containing GWGR motif, few studies have managed to thoroughly examine the disparities between these groups regarding clinical outcomes. Additionally, the results outline a natural history of infection that has not experienced significant advancements during combined antiretroviral therapy (cART). Therefore, this study aimed to conduct a longitudinal assessment in a cohort of patients with HIV-1, investigating the progression of clinical events and laboratory parameters in individuals infected with HIV-1 strains containing GWGR motif and undergoing long-term cART treatment, as well as those without these specific signatures in the V3 region of HIV.

## MATERIALS AND METHODS

### Study design

This study was based on a detailed analysis of the signatures of the V3 loop, spanning amino acids 312-315 of the viral envelope. This study was conducted longitudinally within a cohort monitored for over 23 years, comprising 295 individuals living with HIV. The participants were carefully selected and consistently monitored in the outpatient setting of the Hospital das Clinicas at the Faculty of Medicine, University of Sao Paulo. The study integrated a comparative analysis between the genetic data obtained and various clinical parameters following genetic sequencing. All participants were undergoing cART.

### Amplification and sequencing of the V3 region

Peripheral blood samples from patients were collected during outpatient follow-up and after signing an informed consent form. The RNA from the samples was extracted from plasma using the Qiagen QIAamp Viral RNA Mini kit, following the manufacturer’s instructions. Subsequently, reverse transcription was conducted using the SuperScript III Reverse Transcriptase enzyme (Invitrogen, Carlsbad, CA, USA). A PCR was then performed to amplify a 416-bp fragment at the V3 loop. Samples were sequenced using the BigDye^®^ Terminator v3 Cycle Sequencing kit (Applied Biosystems, Foster City, CA, USA), and the sequences were analyzed on the ABI 3130 model sequencer (Foster City, CA, USA). Sequencer software (Gene Code, version 5.4.6, Ann Arbor, Miami) assembled and edited sequences. Amino acid alignment was conducted using Bioedit software (Carlsbad, CA, USA) based on the reference sequence HXB2 (NC 001802).

### Demographic and laboratory profiles

Demographic data, such as age, gender, and duration of laboratory monitoring, were carefully extracted from the outpatient records of each participant. Specific information, such as CD4+ T cells couting at time points, along with the date of diagnosis and other clinical and laboratory data, was selected via the Laboratory Test Control System (SISCEL), integrated into the Brazilian Unified Health System (SUS). Lastly, time intervals from the first positive test, the initiation of treatment, and the onset of the first AIDS-defining event were calculated to evaluate potential impacts on mortality.

### Statistical analysis

The genetic variability in the V3 region of HIV-1 underwent a descriptive analysis, followed by the categorization of samples into GWGR and antigenically distinct signatures from GWGR. The p-value for categorical variables was determined with the chi-squared test. One-way analysis of variance (ANOVA) with Tukey’s *post-hoc* test was employed to continuous variables exhibiting a normal distribution, while the Kruskal-Wallis’s test was used for nonparametric data. For progression data involving only two variables, the Mann-Whitney’s test was used for nonparametric data, and the unpaired t-test was used for normal distribution. Subsequently, a survival curve was generated between groups to better understand the temporal dimension and disease progression. All analyses were performed using GraphPad Prism software (version 8.0.2, San Diego, CA, USA), with statistical significance established by a 95% confidence interval.

### Ethical considerations

The Ethical Board of the Institute of Tropical Medicine, Faculty of Medicine, University of Sao Paulo, Brazil, approved the study protocol FAPESP process Nº 2011/17881-4, CAPPQESP Nº 0108/08. All participants were aged ≥18 years and provided written consent before study inclusion.

## RESULTS

### Characterization of HIV-1 signatures in the V3 loop

**Figure 1:**
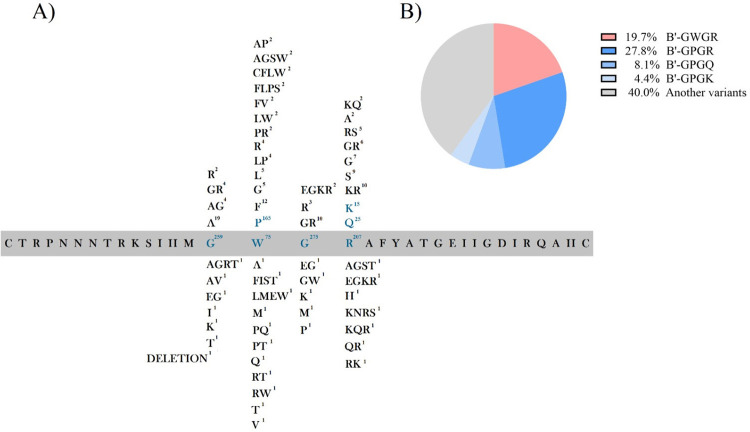
Amino acid is found in the V3 loop of HIV-1: A) Represents the independent analysis of amino acid substitutions in the position 312-315 found in the crown of the V3 loop from env glycoprotein; The blue color represents the region in the GWGR signature and other signature regions analyzed in this study; B) Represents the percentage proportion of identified sequencing instances for GWGR and antigenically distinct signatures.

To characterize HIV-1 signatures, we analyzed amino acid substitutions in positions 312-315 of the envelope protein, which encompasses the V3 loop. Figure 1A presented the frequency of V3 crown positions independently. Due to a substantial number of sequences, we observed significant variability in this viral region encoded by the env gene. However, this observation was anticipated, given the acknowledged diversity in the V3 loop region of the HIV-1 genome. Notably, most of these substitutions resulted from singular mutations (Figure 1A).

Among the analyzed HIV-1 strains, 58 (19.7%) were identified as containing the GWGR motif in the V3 crown, indicating the presence of tryptophan as the second amino acid at position 313 of the envelope glycoprotein (Figure 1B). Among the other strains, 82 (27.8%) presented the GPGR motif, identical to the HXB2 reference strain. Additionally, 24 (8.1%) strains were identified containing glutamine at position 315 (GPGQ), which is identical to HIV-1 ancestral strains. Finally, 13 (4.4%) HIV-1 strains exhibited lysine at position 315, forming the GPGK motif. A total of 118 (40%) HIV-1 strains exhibited different V3 crown motifs from GPGR, GWGR, GPGQ, and GPGK, with low-incidence mutations. Thus, the groups with low incidence were not included in the comparative analysis.

No significant differences were observed among individuals carrying the GWGR and those with antigenically distinct signatures from GWGR regarding gender (*p* = 0.599), age (*p* = 0.8587), and the duration of longitudinal monitoring (*p* = 0.0989) (Table 1). Regarding clinical course of the infection, the CD4+ T cell count from the individual’s latest examination presented no difference between the groups. Neither the CD4+ T cell nadir (minimum count recorded in the medical history) nor the CD4+ T cell zenith (maximum count) showed significant differences between those with the GWGR and those with other V3 crown motifs. Notably, the duration between the first positive test and the occurrence of the initial AIDS-defining event also exhibited no statistically significant difference (*p* = 0.1014). Moreover, none of the V3 crown motifs exhibited a statistically significant association with AIDS-related deaths. However, we must underscore that the cumulative number of total deaths, regardless of their association with AIDS, did not reach statistical significance (*p* = .895), as shown in Table 1.

**Table 1:** Multifactorial analysis of GWGR and non-GWGR signatures.

Characteristic	GWGR (n = 58)	GPGR (n = 82)	GPGQ (n = 24)	GPGK (n = 13)	*p*
Nº female/male subjects^a^	23/35	24/58	7/17	4/9	0.5999
Age, mean years ± SD^b^	55.33 ± 11.71	53.94 ± 10.97	53.57 ± 11,45	55.54 ± 10.93	0.8587
Longitudinal monitoring, mean months ± SD^c^	115.9 ± 21.78	123.3 ± 20.79	115.0 ± 17.21	116.3 ± 24.45	0.0989
Progression to AIDS. No. of non-progression/progression^d^	11/46	21/55	9/15	3/8	0.3799
Time to AIDS progression, mean months ± SD^e^	67 ± 80	60.80 ± 179.6	-	-	0.1014
Absolute CD4+ T cell count, median cells/mm^3 f^	569.0	784.0	748	652.0	0.4185
HIV RNA level, copies/mL^g^	0	0	0	0	0.6243
Nadir, median cells/mm^3 h^	230	244	277	245.5	0.5282
Zenith, median cells/mm^3 i^	955.0	883.5	899.0	1013	0.8711
Total Deaths. Deaths/Living Individuals^j^	10/48	4/78	4/20	1/12	0.0895

The p-value for categorical variables (^a,d,j^) was determined by chi-squared test. One-way ANOVA with Tukey’s *post-hoc* test was applied to continuous variables exhibiting normal distribution (^b^), while the Kruskal-Wallis’s test was used for nonparametric data (^c,f,g,h,i^). The Mann-Whitney’s test was used for progression data involving only two variables (e); ^b^Data were available for 55 individuals with the GWGR signature, 80 with GPGR, 23 with GPGQ, and 13 with GPGK; ^c^Data were available for 55 individuals with the GWGR signature, 79 with GPGR, 22 with GPGQ, and 13 with GPGK; ^d^Data were accessible for 57 individuals with the GWGR signature, 76 with GPGR, 24 with GPGQ, and 11 with GPGK. The progression criterion was set at a CD4+ T cell count threshold below 350 cells per mm^3^; ^e^Data were accessible for 39/57 individuals with the GWGR signature and 54/78 with GPGR. The time elapsed between the date of the first known positive test and the occurrence of the first AIDS-defining event; ^f^Data were accessible for 57 individuals with the GWGR signature, 76 with GPGR, 24 with GPGQ, and 12 with GPGK. The analysis incorporated data from the patient’s latest examination results; ^g^Data was accessible for 57 individuals with the GWGR signature, 76 with GPGR, 24 with GPGQ and 12 with GPGK; ^h,i^Data were accessible for 57 individuals with the GWGR signature, 76 with GPGR, 24 with GPGQ and 12 with GPGK; ^j^Data were accessible for 58 individuals with the GWGR signature, 82 with GPGR, 24 with GPGQ and 13 with GPGK.

Like the overall findings within the women’s group, no significant differences were noted in age (*p* = 0.2832), CD4+ T-cell counts (*p* = 0.3368), nadir (*p* = 0.5478), zenith (*p* = 0.7497), longitudinal monitoring (*p* = 0.6147) or disease progression (*p* = 0.5509), and mortality rates (*p* = 0.1015). However, the mean interval between the first positive HIV test and the manifestation of the initial AIDS-defining event was almost three times longer to the GWGR signature, totaling 89.13 months (± 84.14 SD), compared with those without the GWGR, which recorded 34.74 months (± 49.92 SD) (*p* = 0.0231) (Table 2).

**Table 2:** Multifactorial analysis comparing women with the GWGR signature to non-GWGR

Women	GWGR (n = 23)	non-GWGR (n = 35)	*p*
Age, mean years ± SD^a^	57.14 ± 11.03	55.26 ± 12.63	0.2832
Longitudinal monitoring, mean months ± SD^b^	115.4 ± 21.10	125.7 ± 19.69	0.6147
Progression to AIDS. Nº of non-progression/progression^c^	5/17	10/22	0.5509
Time to AIDS progression, mean months ± SD^d^	89.13 ± 84.14	34.74 ± 49.92	0.0231
Absolute CD4+ T cell count, median cells/mm^3 e^	622.0	807.0	0.3368
HIV RNA level, copies/mL^f^	0	0	0.8499
Nadir, median cells/mm^3 g^	222.5	252.0	0.5478
Zenith, median cells/mm^3 h^	965.0	930.0	0.7497
Total deaths. Deaths/living individuals^i^	5/18	2/33	0.1015

The p-value for continuous and non-parametric data (^a, b, d, e, f, g^) was determined using the Kruskal-Wallis’s test, while the unpaired t-test (^h^) was applied to parametric data. Chi-squared was employed for categorical variables (^c,i^); ^b^Data were accessible for 22 individuals with the GWGR signature and 33 for non-GWGR; ^c^Data were accessible for 22 individuals with the GWGR signature and 32 for non-GWGR; ^d^Data were accessible for 15 individuals with the GWGR signature and 19 for non-GWGR; ^e^Data were accessible for 22 individuals with the GWGR signature and 33 for non-GWGR; ^f^Data were accessible for 22 individuals with the GWGR signature and 33 for non-GWGR; ^g^Data were accessible for 22 individuals with the GWGR signature and 33 for non-GWGR; ^h^Data were accessible for 22 individuals with the GWGR signature and 33 for non-GWGR; ^i^Data were accessible for 23 individuals with the GWGR signature and 35 for non-GWGR.

### Survival disparities post-treatment

**Figure 2:**
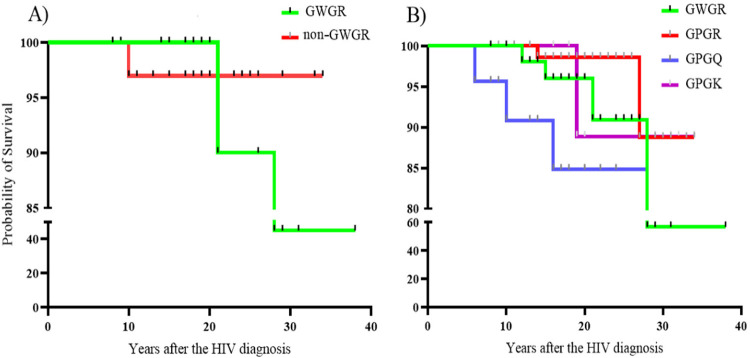
Multifactorial analysis comparing the GWGR to non-GWGR signatures: A) Represents the survival curve for all individuals with the GWGR signature (green) and non-GWGR signatures (GPGR, GPGQ, and GPGK) (red); B) Represents the survival among women with the GWGR signature (green) and those with GPGR (red), GPGQ (blue), and GPGK (purple).

The survival curve analysis did not uncover significant differences between individuals infected with the HIV-1 containing GWGR motif and those harboring viral strains with non-GWGR motifs (Figure 2A). However, the women’s group, characterized by the presence of the GWGR signature, exhibited a comparatively higher survival rate in the early years of cART (Figure 2B). Because of a limited number of women surviving after 20 years of treatment, this rate declined noticeably, dropping to 50%. This figure is notably lower when compared with non-GWGR motifs, which maintained an approximate 90% survival rate.

## DISCUSSION

Over the last few decades, the Brazilian HIV-1 subtype B variant (B’-GWGR) has intrigued retrovirus researchers. Numerous studies have explored how different subtypes of HIV-1 or their signatures can modify the natural course of the infection^8,10,25,27,31^. This particular Brazilian strain has been associated with a decreased rate of progression to an AIDS-defining illness compared with those with the GPGR variant^27^. It has also shown a reduced viral load among female patients and elevated CD4+ T cell counts in this population^8^. The GWGR motif increases the binding avidity of neutralizing antibodies^31^ and does not confer antiretroviral resistance^18^. These findings may contribute to the reported milder pathogenic potential previously observed^33^.

In this context, this study reported that individuals infected with HIV-1 strains containing the GWGR motif at the V3 crown progress more slowly to AIDS disease. However, this effect seems to only be significant among female patients. On average, women with the GPGR strain presented the first AIDS clinical sign approximately three years after the first positive test, whereas patients with the HIV-1 GWGR strain exhibited the first AIDS sign after seven years. This observation aligns with the data obtained from the women’s cohort in this study and corresponds with findings from a previous investigation within our cohort conducted during a pre-cART transitional period^8^.

Women tend to have higher CD4+ T cell counts and lower HIV plasma RNA loads during the asymptomatic phase of infection^34^. Sexual dimorphism in the mammalian immune system has long been recognized in the context of infection with various pathogens, with females often exhibiting more effective immune responses against such threats^35^. Among individuals who demonstrate natural suppression of HIV to undetectable levels without the use of antiretroviral therapy, referred to as elite controllers, women have been identified in some studies to be over-represented^35^. This implies that genetic and immunologic factors likely play a significant role in this population.

The presence of the GWGR motif on HIV strains appears to have played a role in contributing to the extended ‘asymptomatic’ status observed in the female patients participating in this study. The higher permissibility for neutralizing antibodies against V3 containing GWGR, as reported in a previous study^30,31^, can be detrimental to HIV-1 infectivity, effectively blocking the viral cycle. Antibodies that neutralize envelope glycoprotein containing GWGR at V3 loop could potentially prevent pathogenic processes such as cell-cell fusion and CD4+T cell apoptosis. Furthermore, the CCR5 tropism restriction promoted by the GWGR motif^10^ may reduce the HIV-1 cycle and hinder viral progression, considering that, particularly as the disease progresses to advanced stages, coreceptor usage may shift from CCR5 to CXCR4, as previously reported^36^. This switch is often associated with a more aggressive and advanced form of HIV-1 infection^37^, especially when HIV-1 reaches certain reservoirs such as the central nervous system, which require CXCR4 tropism^38^.

Conversely, GPGQ or GPGK motifs favor elevating the pathogenic potential of the HIV-1 strains. For instance, both motifs can reduce the binding capacity of neutralizing antibodies^18^. The higher pathogenic potential of the non-GWGR strains may have contributed to anticipating the HIV-1 disease progression, found by this study. Female patients infected with HIV strains harboring motifs other than GWGR experienced the first AIDS-defining event in less than half the time compared with patients harboring the strain with GWGR. Studies focused on analyzing signatures in position 312-315 of the V3 loop have suggested that specific residues, such as aspartic acid and glutamic acid, may potentially increase viral infectivity. In contrast, hydrophobic amino acids, such as tryptophan, have the potential to significantly reduce HIV-1 adaptability^8,10,11,27^.

Although the progression to AIDS has been slower in patients with HIV-1 GWGR, laboratory data did not confirm that HIV-1 containing GWGR motif holds a milder pathogenic potential compared with strains containing GPGR, GPGQ, and GPGK motifs. For instance, we did not identify significant differences comparing CD4+T cell counts or HIV-1 viral load. In the past, studies have indicated that patients infected with the HIV-1 strain containing the GWGR motif had lower CD4 cell counts and reduced viral loads^8,27,39^. Apparently, individuals infected with HIV-1 strains containing the GWGR motif experienced a more benign impact compared with those infected with strains containing other motifs, such as GPGR or GPGQ. The latter group exhibited a more damaging clinical course of infection, and the therapy effectiveness in addressing these challenges seems to have been limited. As a result, it could not equally impact the clinical course between individuals infected with HIV-1 strains containing the GWGR motif and those with non-GWGR strains.

We must consider that, while antiretroviral therapy played a crucial role in the past, its universal availability was not guaranteed, and various challenges accompanied it. During the period when these analyses were conducted, the landscape of antiretroviral drug use differed significantly. It included delays in initiating therapy, the administration of multiple pills, low antiretroviral therapy adherence, and various adverse effects^40^. However, these challenges have been largely overcome in the current scenario.

Nowadays, HIV-1 causes a chronic illness managed by diverse cART options, allowing the growing population over 50 years to thrive with successful treatment. Adherence to cART is crucial for long-term success. Various antiretroviral drugs targeting different viral replication stages, and new therapies with fewer side effects, represent significant advancements. The introduction of a cost-effective cART regimen (single daily pill, injectables, and drug-eluting implants) enhances HIV-1/AIDS management^39,40^. Thus, aspects related to the natural course of infection described in the literature point towards a slower progression for those related to HIV-1 strain containing GWGR motif^8^, lower AIDS incidence^27^, higher CD4+ T cell counts, and lower RNA viral loads^31^, may be directly associated with a natural infection history before the cART era, while the observed physicochemical aspects^9-11,25^, maybe insufficient currently in the face of new antiretroviral regimens to cause significant differences in the natural history of infection in these individuals.

However, amid theoretical speculations, in other cART periods, the natural evolution of infection showed a more pronounced inclination towards severe clinical manifestations associated with AIDS, often resulting in death^39^. Currently, we must emphasize that with cART evolution, the clinical management of HIV/AIDS has undergone a significant revolution. The cART improvements have allowed more effective and sustained viral suppression and robustly controlled virus replication and contributed to the preservation of CD4 cells and the maintenance of more resilient immune function over time.

## CONCLUSION

Over the 23 years of follow-up in this cohort, 40 deaths occurred among individuals infected with HIV during childhood (PHIV). Note that few of these fatalities were attributed to opportunistic infections or cancer. These data highlight the considerable efficacy of cART in preventing the development of AIDS or death due to immunosuppression in our cohort. However, we must consider that events unrelated to AIDS may have impacted the long-term survival of PHIV in recent years.
